# Cytogenetic and molecular landscape and its potential clinical significance in Hispanic CMML patients from Puerto Rico

**DOI:** 10.18632/oncotarget.27824

**Published:** 2020-11-24

**Authors:** Zeju Jiang, Xinlai Sun, Zhao Wu, Albert Alhatem, Ruifang Zheng, Dongfang Liu, Yaqun Wang, Dibyendu Kumar, Changqing Xia, Bei You, He Wang, Chen Liu, Jie-Gen Jiang

**Affiliations:** ^1^Department of Pathology, Immunology & Laboratory Medicine, Rutgers New Jersey Medical School, Newark, NJ 07103, USA; ^2^Jiangxi Medical College, Nanchang University, Nanchang 330006, Jiangxi, China; ^3^Neogenomics, Carlsbad, CA 92008, USA; ^4^Department of Biostatistics, Rutgers School of Public Health and Rutgers Cancer Institute of New Jersey, New Brunswick, NJ 08901, USA; ^5^Institute of Genomics Medicine, New Jersey Medical School, Rutgers University, Newark, NJ 07103, USA; ^6^Department of Pathology and Laboratory Medicine, Rutgers Robert Wood Johnson Medical School, New Brunswick, NJ 08903, USA; ^7^Department of Pathology, School of Medicine, Yale University, New Haven, CT 06520, USA

**Keywords:** chronic myelomonocytic leukemia, Hispanic, cytogenetic abnormality, gene mutation

## Abstract

Chronic myelomonocytic leukemia (CMML) is a clonal hematopoietic neoplasm that exhibits myelodysplastic and myeloproliferative characteristics with heterogeneous clinical and pathological features. There are limited publications on the ethnic and racial disparity of cytogenetics and genomics in CMML patients. This study aims to define the cytogenetic and molecular landscape in Hispanic CMML patients from Puerto Rico and explore its possible clinical significance. One hundred and eleven (111) Hispanic CMML patients from Puerto Rico were diagnosed in our institute from 2009 to 2018. Karyotypes were available in one hundred and seven (107) patients. Seventeen (17) patients had abnormal karyotypes (17/107, 16%). Compared to previously published data, Hispanic CMML patients in this study had significantly lower rates of overall cytogenetic abnormalities (16% vs 27–28%, *p* < 0.05) and trisomy 8 (2% vs 7%, *p* < 0.05). Among one hundred and eleven (111) Hispanic CMML patients, 40-gene myeloid molecular profile tests were performed in fifty-six (56) CMML patients. Gene mutations were identified in fifty-four (54) patients (96%). The most frequent mutated genes were: *TET2*, *SRSF2*, *ASXL1*, *ZRSR2*, *DNMT3A*, *NRAS*, *CBL*, and *RUNX1*. Twenty-nine (29) out of fifty-six (56) patients (29/56, 52%) had mutated *TET2*/wild type *ASXL1* (*muTET2*/*wtASXL1*). Previous studies indicated that mutated *ASXL1*, *DNMT3A*, *NRAS*, *RUNX1*, and *SETBP1* may associate with an unfavorable prognosis and *muTET2*/*wtASXL1* may associate with a favorable prognosis in CMML patients. Compared to previously published data, Hispanic CMML patients from Puerto Rico in this study had significantly lower mutation rates in *ASXL1* and *SETBP1*, and a higher rate of *muTET2*/*wtASXL1*. The findings raise the possibility of a favorable prognosis in Hispanic CMML patients.

## INTRODUCTION

Chronic myelomonocytic leukemia (CMML) is a clonal hematopoietic malignancy with the presence of sustained monocytosis in peripheral blood alongside myelodysplastic and myeloproliferative characteristics. Its estimated incidence is 4 per 100,000 persons per year. The median age at diagnosis is 71–74 years old. CMML has a propensity for males rather than females, at a ratio of 1.5–3:1 [1]. In the 2008 World Health Organization (WHO) classifications, CMML was divided into 2 subgroups, CMML-1 and CMML-2 [2] while in 2016, it was further classified into 3 subgroups, CMML-0, CMML-1, and CMML-2, according to the percentage of blasts and blast equivalents in bone marrow and peripheral blood [3]. The 2016 WHO classification also recommended categorization of CMML into “proliferative” (MPN-CMML) and “dysplastic” (MDS-CMML) sub-types based on a white blood cell count of ≥ 13 × 10^9^/L for MPN-CMML [3, 4]. In the most recent literature, additional classification of pre-CMML conditions as well as special CMML variants were proposed [5]. The clinical and pathological features of CMML are highly heterogeneous and variable with wide differences in survival and risk of disease evolution into acute myeloid leukemia (AML) or acute myelomonocytic leukemia (AMML).

Clonal cytogenetic abnormalities are found in about 20% to 30% of CMML patients, but none are specific. The common cytogenetic abnormalities include trisomy 8 (+8), loss of the Y chromosome (-Y), abnormalities of chromosome 7 (-7 and 7q-), 20q deletion, trisomy 21 (+21), der(3q), and complex/monosomal karyotypes [6]. These cytogenetic abnormalities are associated with disease risk/prognosis. A step-wise survival analysis resulted in three distinct cytogenetic risk categories: high (complex and monosomal karyotypes), intermediate (all abnormalities excluding high or low risk groups), and low (normal, sole -Y and sole der (3q)) [6]. The CMML specific cytogenetic risk stratification (CPSS) system proposed by Such, E. et al. categorizes patients into three groups: high risk (trisomy 8, chromosome 7 abnormalities, or complex karyotype), intermediate risk (all chromosomal abnormalities excluding high and low risk categories), and low risk (normal karyotype or –Y) [7].

Recurrent somatic mutations have been identified in more than 90% of CMML patients. These mutant genes mainly encode signaling molecules (*NRAS*, *KRAS*, *CBL*, *ETNK1*, *CSF3R*, and *JAK2*), epigenetic regulators (*TET2*, *IDH1*, *IDH2*, *DNMT3A*, *ASXL1*, *SETBP1*, and *EZH2*), splicing factors (*SRSF2*, *SF3B1*, *ZRSR2*, and *U2AF1*), transcription factors (*RUNX1*, *ETV6*, and *NPM1*), and tumor suppressor gene (*TP53*) [1, 3]. Associations between these somatic mutations and disease phenotype or prognosis have been suggested, e.g., co-occurrence of *TET2* and *SRSF2* mutations is common in CMML and specific for myeloid neoplasms with monocytosis [1]; the mutations in *ASXL1*, *NRAS*, *RUNX1*, or *SETBP1* are associated with an unfavorable prognosis [8], whereas mutated *TET2* with wild type *ASXL1* (*muTET2*/*wtASXL1*) is associated with a favorable CMML prognosis [9].

The racial cancer disparities in outcomes have been described and attributed to a combination of biological and nonbiological factors. African Americans continue to have higher cancer mortality rates and shorter overall survival [10]. Age-adjusted overall survival of acute myeloid leukemia (AML) was reported to be worse in Hispanics compared with whites [11]. It was reported that Hispanic whites had an age-adjusted lower incidence rate of CMML compared to non-Hispanic whites [12]. However, there are no published studies on the racial disparity of cytogenetics and genomics in Hispanic CMML patients. The aim of this study is to define the cytogenetic and molecular landscapes of Hispanic CMML patients from Puerto Rico and explore their potential clinical significance. All study methods were carried out in accordance with relevant guidelines and regulations.

## RESULTS

One hundred and eleven (111) Hispanic CMML patients from Puerto Rico were diagnosed in the Genoptix Medical Laboratory from 2009 to 2018. The age range was from 46 to 96 years with a median age of 74. Sixty-five (65) were male and forty-six (46) were female (data not shown). The epidemiological features are similar to the previous published data from CMML patients [1, 3].

Among these patients, karyotype was available in one hundred and seven (107) patients. Ninety (90) patients had normal karyotype (90/107, 84%). Seventeen (17) patients had abnormal karyotype (17/107, 16%): five (5) patients with complex karyotype(s) (5/107, 5%), four (4) patients with –Y (4/107, 4%), two (2) patients with +8 (2/107, 2%), and two (2) patients with -7 or 7q- (2/107, 2%) (Table 1). No cases with 20q deletion, trisomy 21, or sole der (3q) were identified. Compared to previously published data [6, 7], the CMML patients in our study had a significantly lower rate of cytogenetic abnormalities (16% vs 27–28%, *p* < 0.05) (Table 1). The most frequent cytogenetic abnormality in Hispanic CMML patients from Puerto Rico was complex karyotype (5%), followed by -Y (4%), trisomy 8 (2%), and abnormalities of chromosome 7 (2%). Since complex karyotype and abnormalities of chromosome 7 are associated with an unfavorable prognosis, we also compared alone or combined abnormality rates of complex karyotype and abnormalities of chromosome 7 in Hispanic CMML patients from Puerto Rico with these data. Hispanic CMML patients from Puerto Rico had no significant difference in alone or combined rates of complex karyotype and/or abnormalities of chromosome 7 (Table 1).

**Table 1:** Cytogenetic abnormalities in 107 Hispanic CMML patients from Puerto Rico compared with that in Such, E. et al. [7] and Wassie, EA. et al. [6]

Cytogenetic results	Frequency in current study	Frequency in Such, E. et al. [7]	Frequency in Wassie, EA. et al. [6]
Normal karyotype	90/107, 84%	304/414, 73%	294/409, 72%
Abnormal karyotype	17/107, 16%	110/414, 27%^*^ (*p* = 0.0229)	115/409, 28%^*^ (*p* = 0.009)
-Y	4/107, 4%	18/414, 4%	23/409, 6%
+8	2/107, 2%	30/414, 7%^*^ (*p* = 0.0409)	27/409, 6%
-7/7q-	2/107, 2%	6/414, 1%	16/409, 4%
Complex	5/107, 5%	12/414, 3%	13/409, 3%
20q-	0/107	3/414, 1%	9/409, 2%
+21	0/107	NA	9/409, 2%
Sole der (3q)	0/107	NA	10/409, 2%
Other	4/107, 4%	41/414, 10%	37/409, 3%

^*^significant difference (*p* < 0.05) by the Fisher’s test. NA: not available.

Regarding the 2008 WHO subgroup classification [2], we further analyzed the one hundred and seven (107) patients with karyotype results available. Ninety-two (92) patients were diagnosed as CMML-1 (86%) and fifteen (15) as CMML-2 (14%). CMML-1 and CMML-2 rates were similar to that published by Such, E. et al. and Wassie, EA. et al. (Table 2) [6, 7]. There was no significant low rate of CMML-2 in Hispanic CMML patients from Puerto Rico although all these patients (107 patients) had a significantly lower rate of cytogenetic abnormalities. However, Hispanic CMML patients had a significantly lower rate of CMML-1 with abnormal karyotype (Table 2).

**Table 2:** Subtypes in 107 Hispanic CMML patients from Puerto Rico compared with that in Such, E. et al. [7] and Wassie, EA. et al. [6]

Subtype		Frequency in current study	Frequency in Such E. et al. [7]	Frequency in Wassie EA. et al. [6]
CMML-1		92/107, 86%	367/414, 89%	343/409, 84%
CMML-2		15/107, 14%	47/414, 11%	66/409, 16%
CMML-1	Normal Karyotype	82/92, 89%	284/367, 77%	263/343, 77%
	Abnormal Karyotype	10/92, 11%	83/367, 23%^*^ (*p* = 0.0131)	80/343, 23%^*^ (*p* = 0.0087)
CMML-2	Normal Karyotype	8/15, 53%	20/47, 43%	31/66, 47%
	Abnormal Karyotype	7/15, 47%	27/47, 57%	35/66, 53%

^*^significant difference (*p* < 0.05) by the Fisher’s test.

Among the one hundred and eleven (111) Hispanic CMML patients from Puerto Rico diagnosed in our institute from 2009 to 2018, 40-gene myeloid molecular profile tests were performed in fifty-six (56) CMML patients, in which forty-nine (48) patients were diagnosed as CMML-1 and eight (8) as CMML-2. Their ages ranged from 46 to 92 years with a median age of 75. Thirty-five (35) patients were male and twenty-one (21) were female (Supplementary Table 1). The demographic features of these fifty-six (56) Hispanic CMML patients were similar to the published data [3].

**Figure 1:**
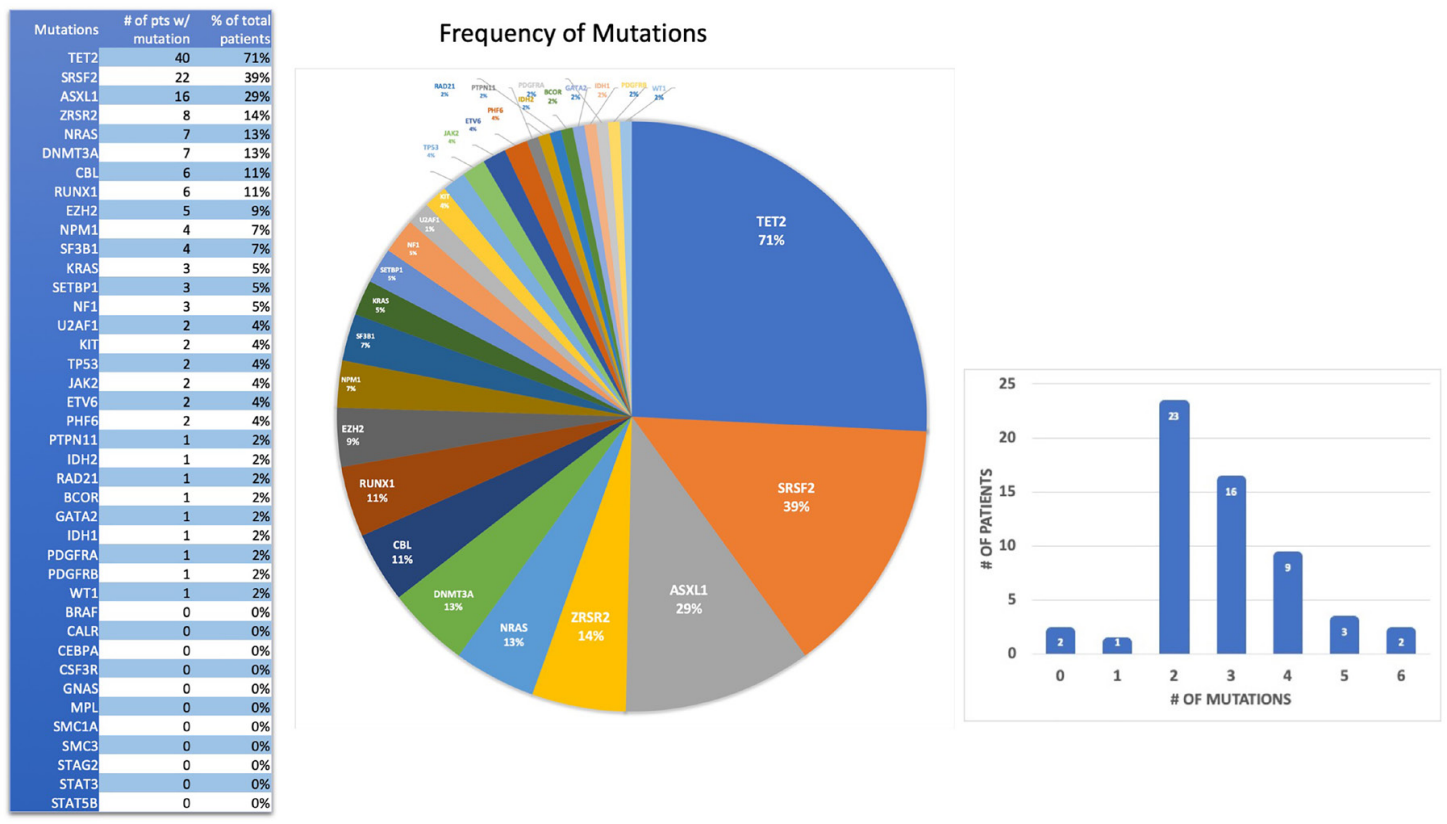
Spectrum and frequency of gene mutations in 56 Hispanic CMML patients.

Fifty-four (54) out of the fifty-six (56) patients had at least one mutation identified (96%) (Table 3): one (1) patient with 1 mutation (1/56) and fifty-three (53) patients with 2 or more mutations (53/56). Most of these patients had 2 to 3 mutations (Figure 1). The mutation frequencies in different genes detected by myeloid molecular profiling tests were: *TET2* (40/56, 71%), *SRSF2* (22/56, 39%), *ASXL1* (16/56, 29%), *ZRSR2* (8/56, 14%), *DNM3A* (7/56, 13%), *NRAS* (7/56, 13%), *CBL* (6/56, 11%), *RUNX1* (6/56, 11%), *EZH2* (5/56, 9%), *NPM1* (4/56, 7%), *SF3B1* (4/56, 7%), *KRAS* (3/56, 5%), *NF1* (3/56, 5%), *SETBP1* (3/56, 5%), *ETV6* (2/56, 4%), *JAK2* (2/56, 4%), *KIT* (2/56, 4%), *PHF6* (2/56, 4%), *TP53* (2/56, 4%), *U2AF1* (2/56, 4%), *BCOR* (1/56, 2%), *GATA2* (1/56, 2%), *IDH1* (1/56, 2%), *IDH2* (1/56, 2%), *PDGFRA* (1/56, 2%), *PDGFRB* (1/56, 2%), *PTPN11* (1/56, 2%), *RAD21* (1/56, 2%), *WT1* (1/56, 2%). The rest of genes in this panel had no mutations detected. The most frequent mutated genes were: *TET2*, *SRSF2*, *ASXL1*, *ZRSR2*, *DNMT3A*, *NRAS*, *CBL*, and *RUNX1* (> 10%, see Figure 1).

**Table 3:** Mutation rate detected by myeloid molecular panel tests in 56 Hispanic CMML patients from Puerto Rico

With mutation(s)	Without mutation(s)
54/56, 96%	2/56, 4%

**Figure 2:**
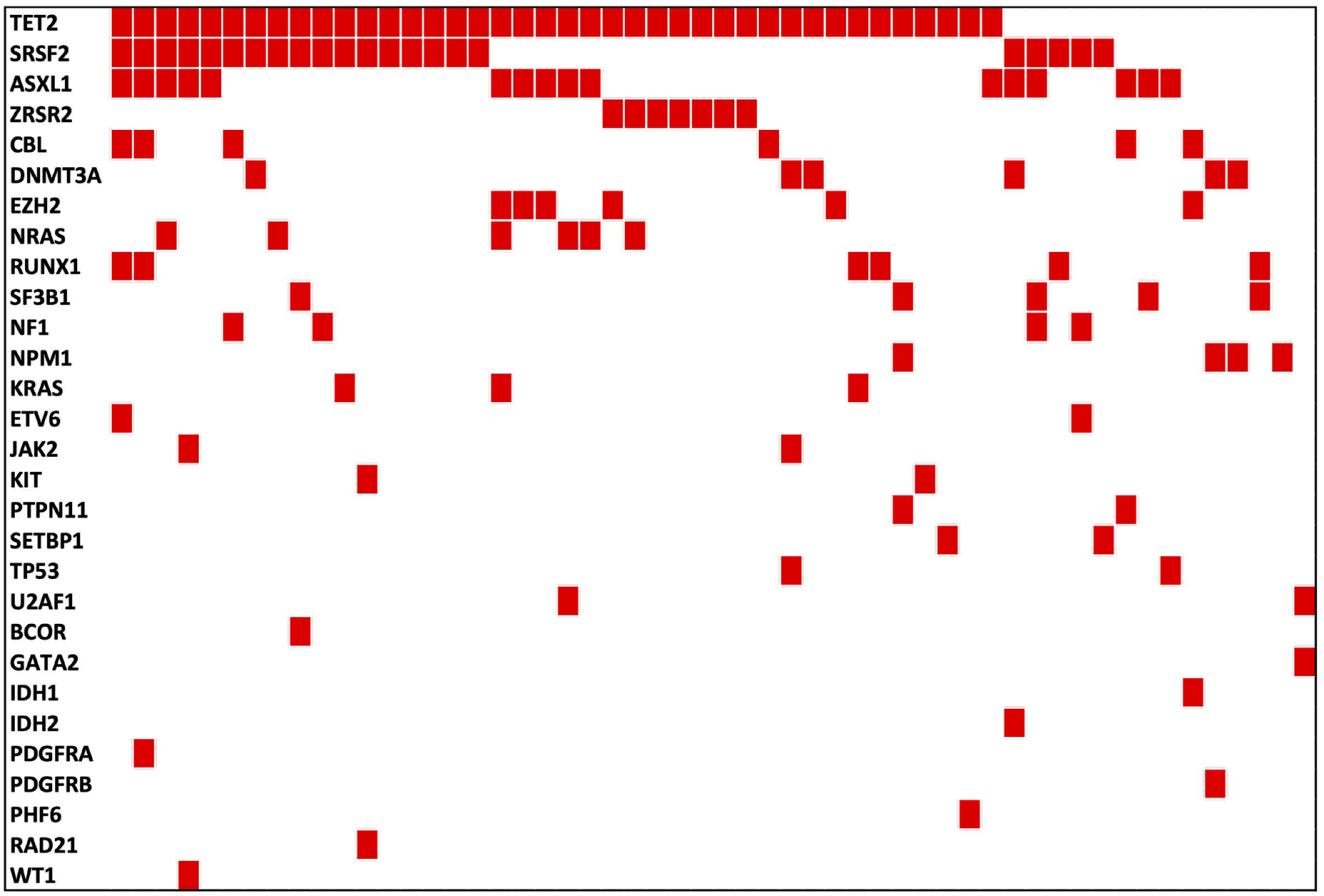
Gene mutation distribution in Hispanic CMML patients from Puerto Rico.

Data in Supplementary Table 1, Supplementary Table 2, Figure 2 and Table 4 summarized the gene mutation frequency in CMML patients in this study in comparison with two other large-scale studies. Our results showed that epigenetic regulator *TET2* gene was the most common mutated gene (40/56, 71%) which is similar to previous studies. Table 5 shows the comparison of *muTET2*/*wtASXL1* rates in our fifty-six (56) Hispanic CMML patients from Puerto Rico with two previously published studies [8, 9]. In our current study, twenty-nine (29) out of fifty-six (56) patients (~52%) had *muTET2*/*wtASXL1* (Table 5). Compared with the data published by Patnaik, MM. et al. [9], Hispanic CMML patients in this study had significantly lower mutation rates in *ASXL1* (*p* = 0.0196) and *SETBP1* (*p* = 0.0183); but had significantly higher mutation rates in *TET2* (*p* = 0.0011), *ZRSR2* (*p* = 0.035), *EZH2* (*p* = 0.0101), and a higher rate of *muTET2*/*wtASXL1* (*p* = 0.0000) (Tables 4 and 5). Compared with the data published by Elena, C. et al. [8], Hispanic CMML patients in this study had significantly higher mutation rates in *TET2* (*p* = 0.0005), *ZRSR2* (*p* = 0.011), *DNMT3A* (*p* = 0.0187) and *muTET2*/*wtASXL1* (*p* = 0.0000) (Tables 4 and 5). There was no significant difference in mutation rate of *NRAS* (Table 4). In our study there were four (4) CMML patients harboring *NPM1* mutations (4/56, 7%). One patient was diagnosed as CMML-2 and the other three (3) were diagnosed as CMML-1 with no identifiable morphological, immunophenotypic, and immunohistochemical evidence of evolving AML (Supplementary Table 3).

**Table 4:** Frequency comparison of gene mutations in CMML patients

Gene	Current study	Elena, C. et al. [8]	*p* value	Patnaik, MM. et al. [9]	*p* value
TET2^**^	40/56 (71%)	95/214 (44%)	0.0005	80/175 (46%)	0.0011
SRSF2	22/56 (39%)	83/214 (39%)	Ns	93/175 (53%)	Ns
ASXL1^**^	16/56 (29%)	79/214 (37%)	Ns	82/175 (47%)	0.0196
ZRSR2	8/56 (14%)	9/214 (4%)	0.011	9/175 (5%)	0.0359
DNMT3A^**^	7/56 (13%)	8/214 (4%)	0.0187	9/175 (5%)	Ns
NRAS^**^	7/56 (13%)	25/214 (12%)	Ns	21/175 (12%)	Ns
CBL	6/56 (11%)	18/214 (8%)	Ns	25/175 (14%)	Ns
RUNX1^**^	6/56 (11%)	17/214 (8%)	Ns	25/175 (14%)	Ns
EZH2	5/56 (9%)	15/214 (7%)	Ns	2/175 (1%)	0.0101
NPM1	4/56 (7%)	NA		5/175 (3%)	Ns
SF3B1	4/56 (7%)	12/214 (6%)	Ns	10/175 (6%)	Ns
KRAS	3/56 (5%)	19/214 (9%)	Ns	NA	
NF1	3/56 (5%)	7/214 (3%)	Ns	NA	
SETBP1^**^	3/56 (5%)	19/214 (9%)	Ns	33/175 (19%)	0.0183
ETV6	2/56 (4%)	NA		NA	
JAK2	2/56 (4%)	15/214 (7%)	Ns	7/175 (4%)	Ns
KIT	2/56 (4%)	5/214 (2%)	Ns	2/175 (1%)	Ns
PHF6	2/56 (4%)	NA		NA	
TP53	2/56 (4%)	NA		9/175 (5%)	Ns
U2AF1	2/56 (4%)	9/214 (4%)	Ns	14/175 (8%)	Ns
BCOR	1/56 (2%)	NA		NA	
GATA2	1/56 (2%)	NA		NA	
IDH1	1/56 (2%)	NA		NA	
IDH2	1/56 (2%)	12/214 (6%)	Ns	8/175 (5%)	Ns
PDGFRA	1/56 (2%)	NA		NA	
PDGFRB	1/56 (2%)	NA		NA	
PTPN11	1/56 (2%)	5/214 (2%)	Ns	8/175 (5%)	Ns
RAD21	1/56 (2%)	NA		NA	
WT1	1/56 (2%)	NA		NA	
CEBPA	0/56	NA		11/175 (6%)	Ns
SH2B3	NA	NA		8/175 (5%)	
CSF3R	0/56	NA		3/175 (2%)	Ns
IDH1	0/56	NA		3/175 (2%)	Ns
SUZI12	NA	NA		2/175 (1%)	
CALR	0/56	NA		1/175 (1%)	Ns
FLT3	NA	NA		1/175 (1%)	
MPL	0/56	NA		0/175	
IKZF	NA	NA		0/175	
CUX1	NA	8/214 (4%)		NA	
EP300	NA	7/214 (3%)		NA	
ETNK1	NA	7/214 (3%)		NA	

^**^prognosis-associated gene mutations. *P* value by the Fisher’s test. Ns: no significance according to Benjamini–Hochberg procedure. NA: not available.

**Table 5:** Comparison of *muTET2/wtASXL1* rates in 56 Hispanic CMML patients from Puerto Rico with previous published data

*muTET2*/*wtASXL1* in current study	*muTET2*/*wtASXL1* in Elena C. et al. [8]	*muTET2*/*wtASXL1* in Patnaik MM. et al. [9]
29/56, 52%	45/214, 21% (*p* = 0.0000)	38/175, 22% (*p* = 0.0000)

*P* value by the Fisher’s test.

## DISCUSSION

Ethnic and racial disparities have been described in the outcomes of hematological malignancies such as acute leukemia and attributed to a combination of biological and non-biological factors [13, 14]. Age-adjusted overall survival of AML was reported to be worse in Hispanics compared with whites [11]. It was demonstrated that cytogenetic abnormalities are common in AML and are associated with a significant prognostic impact on AML patients. African Americans were more commonly classified in the favorable and unfavorable cytogenetic risk groups, and less commonly classified in the intermediate group than whites [15]. AML appears less common in Hispanics when compared with whites; however, acute promyelocytic leukemia (APL) appears comparatively more common in Hispanics when compared with whites [16]. It was revealed that there were significantly higher mutation rates of *ASXL1* and *TET2* genes in Hispanic AML patients than in white AML patients, which may provide a biological explanation for the inferior outcomes of AML in Hispanics [11]. The varied distribution of acute leukemia among these ethnic groups suggests that host susceptibility factors are critical determinants of disease in one group, but not in another group [11]. The extent to which the environment interacts with these factors is unknown. Although there are limited published data, it has been shown that African American patients with MDS had worse overall survival (OS) compared to whites. African American patients are more likely to have poor-risk cytogenetics and high- or very-high-risk categories per IPSS-R, and a higher incidence of poor-risk mutations such as *TP53* [17].

It was reported that Hispanic whites had an age-adjusted lower incidence rate of CMML compared to non-Hispanic whites [12]. However, there are no published studies on the outcome disparity in Hispanic CMML patients. Our current study attempts to define the cytogenetic and molecular landscapes of Hispanic CMML patients from Puerto Rico and explore their potential clinical significance. This study reveals, for the first time, that Hispanic CMML patients from Puerto Rico had different patterns of cytogenetic and molecular abnormalities. The findings raise a possibility of a better prognosis in Hispanic CMML patients from Puerto Rico.

The demographic features of one hundred and seven (107) Hispanic CMML patients with karyotype results available and that of the fifty-six (56) Hispanic CMML patients with 40-gene myeloid molecular panel performed were similar to the published data [3], which indicate that our current investigation is representative.

Hispanic CMML patients from Puerto Rico showed varied cytogenetic abnormalities. Trisomy 8, abnormalities of chromosome 7, loss of the Y chromosome, and complex karyotype were the most prevalent chromosomal abnormalities in this study. In the studies by Such, E. et al. [7] and Wassie, EA. et al. [6], however, +8 was most common, followed by -Y and -7/7q-, and complex karyotype constituted 3% of the total patients (Table 1). CMML Hispanic patients from Puerto Rico had a significantly lower rate of overall cytogenetic abnormalities in our current investigation. Compared with the data published by Such, E. et al. [7], trisomy 8 rate was significantly lower in Hispanic CMML patients in our study. Similar to previous studies, Hispanic CMML-2 had significantly more patients with cytogenetic abnormalities than Hispanic CMML-1. However, these CMML patients had a significantly lower rate of CMML-1 with abnormal karyotype (Table 2).

Sole trisomy 8 is not considered presumptive evidence of myelodysplastic syndrome (MDS) in cases without morphological evidence of dysplasia. This is in part because trisomy 8 can be identified as a constitutional trisomy 8 mosaicism (cT8M) [18].

The association between the presence of a cT8M and increased risk of developing Behçet syndrome [19] as well as a high risk of developing myeloid neoplasms [20] have already been demonstrated. As in IPSS of MDS, trisomy 8 was considered among the intermediate risk cytogenetic abnormalities in CMML [6]; furthermore, that aberration was included in the high-risk cytogenetic category of the new CMML-specific cytogenetic risk classification by Such, E. et al [7]. The underlying reason of a lower rate of +8 in Hispanic CMML patients from Puerto Rico is uncertain. It is possible that the healthy Hispanic population from Puerto Rico may have a relatively lower rate of cT8M, which leads a lower rate of +8 in Hispanic CMML patients from Puerto Rico.

Gene mutations were detected in more than 90% of CMML patients. These mutations commonly involve the following categories: epigenetic regulator genes, chromatin regulation and histone modification genes, splicing machinery genes, cohesin complex genes, DNA damage response genes, and signal transduction and tyrosine kinase pathway genes [1]. Recent studies suggested that the preferred order of mutation accumulation is epigenetic control gene mutations first, spliceosome component mutations next, followed by transcription factor mutations and then signal pathway gene mutations. Epigenetic regulators are the most commonly mutated genes in CMML patients [1]. Our current study showed similar features (Table 4, Figure 2 and Supplementary Table 2). Epigenetic regulator *TET2* gene was the most common mutated gene (40/56, 71%).

Overall mutation rates detected by 40-gene myeloid molecular panel in present study were similar to that in these previous studies [8, 9]. Ninety-six percent of Hispanic CMML patients in Puerto Rico harbor at least one mutation. It was suggested that somatic mutations in *ASXL1*, *RUNX1* and *SETBP1* as well as *RAS* pathway mutations had significant independent negative prognostic impact on CMML patients [21, 22]. Mutations in *TET2*, *ASXL1*, *DNMT3A*, *NRAS*, *RUNX1*, and *SETBP1* genes may associate with CMML prognosis. Studies indicated that *ASXL1*, *DNMT3A*, *NRAS*, *RUNX1*, and *SETBP1* mutations are associate with an unfavorable prognosis in CMML patients [1, 8]. Mutated *TET2* with wild type *ASXL1* (*muTET2*/*wtASXL1*) is associated with a favorable prognosis [9]. We compared our results from Hispanic CMML patients from Puerto Rico with the above two previous published studies. Both studies had relatively large patient population with CMML and large gene panels applied in their studies [8, 9]. Hispanic CMML patients from Puerto Rico in our current study had lower mutation rates in *ASXL1* and *SETBP1*, but a higher mutation rate of *DNMT3A*. *DNMT3A* mutation was reported to be associated with an unfavorable prognosis in CMML [23], the true clinical significance of *DNMT3A* mutation in Hispanic CMML patients and how *DNMT3A* interacts with other mutations are uncertain.

TET2 catalyzes demethylation and upregulates transcription through conversion of 5-methyl-cytosine to 5-hydroxymethyl-cytosine. *TET2* mutations are common and thought to be the driver mutations in CMML [24]. The prognostic relevance of *TET2* mutations is uncertain with some studies demonstrating controversary impact on overall survival [25]. *ASXL1* mutations *in vitro* studies could enhance the de-ubiquitinase activity of the ASXL1–BAP1 (BRCA associated protein 1) complex, which then may cooperate with loss of *TET2* to skew towards myeloid development [26]. Recent studies revealed a favorable impact from *TET2* mutations in the absence of *ASXL1* mutations [9]. Our current observation indicates that Hispanic CMML patients have a significantly higher rate in *muTET2*/*wtASXL1*.

It was suggested that most *NPM1* mutations in CMML patients likely indicated disease progression to acute myeloid leukemia [27]. There were four (4) CMML patients harboring *NPM1* mutations (4/56, 7%) in this study. One patient (1) was diagnosed as CMML-2 while the other three (3) were diagnosed as CMML-1 (Supplementary Table 3). Follow-up with these patients may provide more valuable information regarding disease progression to AML.

Due to overall a low rate of cytogenetic abnormalities in Hispanic CMML patients from Puerto Rico, the correlation between cytogenetic and molecular abnormalities in this population was not assessed.

In summary, we examined cytogenetic abnormalities and mutation frequencies in one hundred seven (107) and fifty-six (56) Hispanic CMML patients from Puerto Rico, respectively. These CMML patients had a significantly lower rate in cytogenetic abnormalities, significantly lower mutational rates in *ASXL1* and *SETBP1*, and a significantly higher rate in *muTET2*/*wtASXL1*. Since the cytogenetic and molecular profiles were suggested to be prognosis-associated, our current cytogenetic and molecular profiling data in Hispanic patients from Puerto Rico raise a possibility of a better prognosis in Hispanic CMML patients. To our best knowledge, this is a first study of cytogenetic and molecular abnormalities and their potential clinical significance in Hispanic CMML patients. It is uncertain if Hispanic CMML patients from other areas in the United States have similar cytogenetic and molecular features. Further studies are warranted to clarify this phenomenon.

## MATERIALS AND METHODS

### Patients

We conducted a retrospective investigation of Hispanic CMML patients from Puerto Rico diagnosed in Genoptix Medical Laboratory in Carlsbad, California, between 2009 and 2018. The specimens were submitted to Genoptix Medical Laboratory randomly. The patients were from twenty (20) medical centers or doctor offices across whole territory of Puerto Rico, which likely represents the Hispanic population in Puerto Rico. IRB approval to perform a retrospective chart review to collect and analyze clinical data, including laboratory, cytogenetic, mutational, demographic and pathological diagnostic data, was issued by Sterling IRB (ID: 6173) and informed consent was waived by this IRB. The diagnosis of CMML was established according to the criteria proposed by 2008 World Health Organization (WHO) [2].

### Karyotype analysis

Karyotype studies were performed according to the established standard protocol in Genoptix Medical Laboratory. Briefly, bone marrow aspirate cells were cultured for 24 and 48 hours without stimulation. G-banded metaphase cells were prepared by using standard techniques. When successful cell cultures were achieved, at least 20 metaphases with good banding were analyzed for each sample. A clonal abnormality was defined as the same numerical gain or structural abnormalities in at least 2 metaphase cells or the same numerical loss in at least 3 metaphase cells. A complex karyotype was defined as 3 or more chromosome abnormalities. The karyotypes were recorded by following the recommendations in the International System for Human Cytogenetic Nomenclatures (ISCN 2008).

### Myeloid molecular profile

Myeloid Molecular Profile tests of 40 genes were performed in Genoptix Medical Laboratory in Carlsbad, California, on an Illumina MiSeq instrument. Patient genomic DNA was isolated from bone marrow aspirates or peripheral blood and utilized to identify relevant single nucleotide variants (SNV), insertion/deletions (Indel), and copy number variations (CNV). The DNA sequence of targeted regions of the *ASXL1, BCOR, BRAF, CALR, CBL, CEBPA, CSF3R, DNMT3A, ETV6, EZH2, GATA2, GNAS, IDH1, IDH2, JAK2, KIT, KRAS, MPL, NF1, NPM1, NRAS, PDGFRA, PDGFRB PHF6, PTPN11, RAD21, RUNX1, SETBP1, SF3B1, SMC1A, SMC3, SRSF2, STAG2, STAT3, STAT5B, TET2, TP53, U2AF1, WT1, ZRSR2* genes was determined using an amplicon-based targeted next-generation sequencing (NGS) technology. The genomic alterations within each of these genes were analyzed through proprietary bioinformatic software and interpreted in conjunction with reference databases such as COSMIC, ClinVar, gnomAD, and dbSNP. Quality control metrics include a minimum input of 20 ng, with an optimal input of 100 ng of genomic DNA, and average mean sequencing depth of 500× coverage. The limits of detection (LOD) are 5% for SNV, 10% for Indels, ≥ 6 copies for gene amplifications, and ≤ 0.3 copies for homozygous gene deletions. Insertions greater than 15 nucleotides and deletions greater than 52 nucleotides may not be detected. Benign sequence variants are not reported.

### Statistical analysis

The Fisher’s exact test was used to analyze differences in the distribution of cytogenetic and molecular abnormalities among different CMML patient populations. Multiple comparison correction was performed to control false discovery rate (FDR) using Benjamini–Hochberg procedure and then significance was decided accordingly.

### Ethics approval and consent to participate

The study has been examined and certified by the Ethics Committee of Sterling IRB (ID:6173) in agreement with institutional guidelines.

### Availability of data and materials

The datasets used or analyzed during the current study are available from the corresponding author on reasonable request.

## SUPPLEMENTARY MATERIALS



## Abbreviations

CMML: chronic myelomonocytic leukemia
AML: acute myeloid leukemia
AMML: acute myelomonocytic leukemia
MDS: myelodysplastic syndrome
