# Supplementary material for: Cytogenetic and molecular landscape and its potential clinical significance in Hispanic CMML patients from Puerto Rico
Source: Oncotarget. 2020 Nov 24;11(47):4411–20. doi: 10.18632/oncotarget.27824 (PMC7720771; doi:10.18632/oncotarget.27824)
Supplement: Supplementary file 1 [file oncotarget-11-4411-s001.pdf]

Cytogenetic and molecular landscape and its potential clinical significance in Hispanic CMML patients from Puerto Rico

SUPPLEMENTARY MATERIALS

Supplementary Table 1: Mutation distribution in 56 Hispanic CMML patients

Patient#	Age	Gender	Diagnosis	Mutations Detected by Myeloid Molecular Profile
1	69	M	CMML1	ASXL1 and TP53
2	77	M	CMML1	EZH2, ASXL1, and TET2
3	65	F	CMML1	NPM1
4	89	F	CMML1	PHF6 and TET2
5	79	M	CMML1	SF3B1 and TET2
6	79	F	CMML1	ASXL1, IDH2, SRSF2, and DNMT3A
7	83	F	CMML1	ASXL1, NRAS, TET2, and U2AF1
8	79	M	CMML1	ASXL1, SRSF2, and TET2
9	76	F	CMML1	ASXL1, TET2, and EZH2.
10	70	M	CMML1	CBL, ETV6, SRSF2, ASXL1, TET2, and RUNX1
11	46	M	CMML2	No mutations
12	47	F	CMML1	DNMT3A and NPM1
13	69	F	CMML1	DNMT3A, NPM1, and PDGFRB
14	62	F	CMML1	DNMT3A, SRSF2, and TET2
15	66	F	CMML2	DNMT3A, TP53, JAK2, and TET2
16	85	M	CMML2	EZH2, CBL, and IDH1
17	63	M	CMML1	KIT, RAD21, SRSF2, and TET2.
18	73	F	CMML1	KRAS, NRAS, ASXL1, EZH2, and TET2
19	66	F	CMML1	KRAS, RUNX1, and TET2
20	82	M	CMML1	KRAS, SRSF2, and TET2
21	74	M	CMML2	NPM1, PTPN11, SF3B1, TET2, and DNMT3A
22	68	F	CMML1	NRAS, ASXL1, and TET2
23	74	M	CMML2	RUNX1 and SF3B1
24	73	M	CMML1	SETBP1, SRSF2, and ZRSR2
25	86	M	CMML1	SF3B1 and ASXL1
26	82	M	CMML1	SRSF2 and RUNX1
27	81	M	CMML1	SRSF2 and TET2
28	75	F	CMML1	SRSF2 and TET2
29	67	M	CMML1	SRSF2 and TET2
30	80	F	CMML1	SRSF2, TET2, and NF1
31	69	M	CMML1	TET2 and DNMT3A
32	70	F	CMML1	TET2 and KIT
33	89	M	CMML1	TET2 and ZRSR2
34	82	M	CMML1	TET2, ASXL1, NRAS, and SRSF2

35	80	M	CMML2	U2AF1 and GATA2
36	67	M	CMML1	ZRSR2 and TET2
37	71	M	CMML1	ASXL1, CBL, SRSF2, TET2, PDGFRA, and RUNX1
38	67	M	CMML1	ASXL1, JAK2, SRSF2, TET2, and WT1
39	79	M	CMML1	ASXL1, NF1, SETBP1, and SRSF2
40	74	F	CMML2	ASXL1, PHF6, and CBL
41	80	F	CMML1	ASXL1, TET2, and NRAS
42	78	M	CMML1	CBL and TET2
43	70	F	CMML1	CBL, SRSF2, TET2, and NF1
44	92	F	CMML1	NF1, SRSF2, and ETV6
45	70	F	CMML1	No mutations
46	72	M	CMML1	NRAS, SRSF2, and TET2
47	70	M	CMML2	NRAS, TET2, and ZRSR2
48	78	M	CMML1	RUNX1 and TET2
49	87	M	CMML1	SRSF2 and TET2
50	54	M	CMML1	SRSF2 and TET2
51	88	M	CMML1	SRSF2, TET2, BCOR, and SETBP1
52	76	F	CMML1	TET2 and EZH2
53	71	M	CMML1	TET2 and ZRSR2
54	78	M	CMML1	TET2 and ZRSR2
55	67	M	CMML1	TET2 and ZRSR2
56	89	M	CMML1	TET2, ZRSR2, and EZH2

Supplementary Table 2: Major classes of gene mutation in Hispanic CMML patients

Major class of gene mutation	Gene	Frequency of mutation
Epigenetic Control	TET2	40/56 (71%)
	ASXL1	16/56 (29%)
	DNMT3A	7/56 (13%)
	EZH2	5/56 (7%)
	SETBP1	3/56 (5%)
	IDH1	1/56(2%)
	IDH2	1/56 (2%)
	RAD21	1/56 (2%)
Cell signaling	NRAS	7/56 (13%)
	CBL	6/56 (11%)
	KRAS	3/56 (5%)
	NF1	3/56 (5%)
	JAK2	2/56 (4%)
	KIT	2/56 (4%)
	PDGFRA	1/56 (2%)
	PDGFRB	1/56 (2%)
	PTPN11	1/56 (2%)
RNA splicing	SRSF2	22/56 (39%)
	ZRSR2	8/56 (14%)
	SF3B1	4/56 (7%)
	U2AF1	2/56 (4%)
Transcriptional regulation	NPM1	4/56 (7%)
	RUNX1	6/56 (11%)
	ETV6	2/56 (4%)
	TP53	2/56 (4%)
	PHF6	2/56 (4%)
	BCOR	1/56 (2%)
	GATA2	1/56 (2%)
	WT1	1/56 (2%)

Supplementary Table 3: Hispanic CMML patients with mutated NPM1

Case[#]	Age (years)/Gender	Karyotype	Positive mutations	Diagnosis
1	65/F	46, XX	NPM1	CMML-1
2	74/M	46, XY	DNMT3A, NPM1, PTPN11, SF3B1, TET2	CMML-2
3	47/F	46, XX	DNMT3A, NPM1	CMML-1
4	69/F	46, XX	DNMT3A, NPM1, PDGFRB	CMML-1
